# Regulation of glucose uptake in lymphoma cell lines by c-MYC- and PI3K-dependent signaling pathways and impact of glycolytic pathways on cell viability

**DOI:** 10.1186/s12967-017-1258-9

**Published:** 2017-07-19

**Authors:** Martina Broecker-Preuss, Nina Becher-Boveleth, Andreas Bockisch, Ulrich Dührsen, Stefan Müller

**Affiliations:** 1 Department of Nuclear Medicine, University Hospital Essen, Hufelandstr. 55, 45122 Essen, Germany; 2 Department of Hematology, University Hospital Essen, Hufelandstr. 55, 45122 Essen, Germany; 3 Department of Clinical Chemistry, University Hospital Essen, Hufelandstr. 55, 45122 Essen, Germany; 4 Institute of Pathology, University Hospital Essen, Hufelandstr. 55, 45122 Essen, Germany

**Keywords:** Warburg effect, Glucose metabolism, Lymphoma, ^18^F-fluoro-2-deoxy-d-glucose, 2-deoxy-d-glucose

## Abstract

**Background:**

Changes in glucose and energy metabolism contribute to the altered phenotype of cancer cells and are the basis for positron emission tomography with ^18^F-fluoro-2-deoxy-d-glucose (FDG) to visualize tumors in vivo. The molecular background of the enhanced glucose uptake and its regulation in lymphoma cells is not fully clarified and may provide new possibilities to reverse the altered metabolism. Thus in this study we investigated regulation of glucose uptake by different signaling pathways. Furthermore, the effect of the glucose analog 2-deoxy-d-glucose (2-DG) alone and in combination with other inhibitors on cell survival was studied.

**Methods:**

An FDG uptake assay was established and uptake of FDG by lymphoma cells was determined after incubation with inhibitors of the c-MYC and the PI3K signalling pathways that are known to be activated in lymphoma cells and able to regulate glucose metabolism. Inhibitors of MAPK signalling pathways whose role in altered metabolism is still unclear were also investigated. Expression of mRNAs of the glucose transporter 1 (GLUT1), hexokinase 2 (HK2), glucose-6-phosphatase (G6Pase) and lactate dehydrogenase A (LDHA) and of the glucose metabolism-regulating micro RNAs (miRNA) miR21, -23a, -133a, -133b, -138-1 and -143 was determined by RT-PCR. Cell viability was analysed by MTT assay.

**Results:**

Treatment with the c-MYC inhibitor 10058-F4 and inhibitors of the PI3K/mTOR pathway diminished uptake of FDG in all three cell lines, while inhibition of MAPK pathways had no effect on glucose uptake. Expression of glycolysis-related genes and miRNAs were diminished, although to a variable degree in the three cell lines. The c-MYC inhibitor, the PI3K inhibitor LY294002, the mTOR inhibitor Rapamycin and 2-DG all diminished the number of viable cells. Interestingly, in combination with 2-DG, the c-MYC inhibitor, LY294002 and the p38 MAPK inhibitor SB203580 had synergistic effects on cell viability in all three cell lines.

**Conclusions:**

c-MYC- and PI3K/mTOR-inhibitors decreased viability of the lymphoma cells and led to decreased glucose uptake, expression of glycolysis-associated genes, and glucose metabolism-regulating miRNAs. Inhibition of HK by 2-DG reduced cell numbers as a single agent and synergistically with inhibitors of other intracellular pathways. Thus, targeted inhibition of the pathways investigated here could be a strategy to suppress the glycolytic phenotype of lymphoma cells and reduce proliferation.

## Background

Altered metabolism is a characteristic of most cancer cells and contributes to the needs of transformed cells for rapid proliferation, enhanced energy turnover, anabolic pathways and regulation of redox potential [1, 2]. Both normal and tumor cells use glucose and glutamine as substrates for ATP synthesis and production of components of cellular metabolism like amino acids, nucleosides and fatty acids [3]. Non-transformed cells predominantly use glycolysis and the TCA cycle to metabolize glucose to CO_2_ and H_2_O. Cancer cells on the other hand take up larger amounts of glucose compared normal cells and metabolize it through glycolysis at higher rates [3]. The pyruvate produced by glycolysis in cancer cells is preferentially metabolized to lactate by cytoplasmic lactate dehydrogenases [4]. This shift from ATP production through oxidative phosphorylation to ATP generation through glycolysis is called “aerobic glycolysis” or the “Warburg effect”. It occurs even in the presence of sufficient oxygen and was first described by Otto Warburg [5].

Based on the prevalence of the enhanced glucose uptake and metabolism in cancer cells, positron emission tomography (PET) with the glucose analogue ^18^F-fluoro-2-deoxy-d-glucose (FDG) is a non-invasive molecular imaging tool to detect many tumor entities and monitor therapeutic response by analysing tumor metabolic activity [4, 6]. FDG, similar to glucose, is transported into the cell via glucose transporters (GLUTs) and is phosphorylated by hexokinase (HK) to FDG-6-phosphate. FDG-6-phosphate is trapped in the cell since it is no substrate for glycolytic or pentose phosphate pathways, unable to diffuse out of the cell and it is dephosphorylated slowly. Thus, FDG accumulates in the cell at a rate proportional to glucose utilization and tumors with an increased uptake of glucose can be visualized non-invasively by PET which also enables quantitative assessment of glucose utilization [7, 8].

Metabolic changes in cancer were considered as a secondary effect of the transformation process. Molecular mechanisms that lead to metabolic reprogramming and to the metabolic phenotype of cancer cells are only partly understood and include activation of oncogenes and kinases like c-MYC, members of the phosphoinositide 3-kinase (PI3K) signalling pathway [PI3K, protein kinase B (AKT), mammalian target of rapamycin (mTOR)], stabilization of transcription factors like hypoxia-inducible factor 1 (HIF-1) and inactivation of tumor suppressor genes like p53 [3, 4, 9]. Concordant with the increased uptake of glucose, expressions of GLUT genes, namely GLUT1 and genes of glycolytic enzymes are increased in many cancers [Review: 10]. Besides transcription factors, gene expression is regulated also by microRNAs (miRNAs) which are endogenous small non-coding RNAs of 18–25 nucleotides and regulate gene expression by binding to the 3′-untranslated region of the target mRNA [11]. Depending on their target genes, miRNA can function as tumor-suppressors or oncogenic miRNAs. A few specific miRNAs are described to be involved in glucose metabolism in cancer cells including miR21, -23a,- 133a, -133b, -138-1 and -143 [12–14].

Non-Hodgkin’s lymphoma (NHL) is a heterogeneous group of diseases and includes several lymphoma subtypes like diffuse large B-cell lymphoma (DLBCL), Burkitt lymphoma, mantle cell lymphoma, follicular lymphoma and others [15]. NHLs display enhanced FDG uptake and thus PET is used for staging, monitoring of therapy response and prognostication of these patients. High glucose uptake is seen as a surrogate marker of an aggressive tumor and associated with poor outcome in DLBCL [16, 17]. Patients with NHL are usually treated with a chemotherapeutic regime including rituximab, cyclophosphamide, doxorubicin, vincristine and prednisone (R-CHOP). However, only in 40–50% of patients a complete response is achieved after first therapy [18]. Interim PET after a few cycles of chemotherapy may be used according to current guidelines for NHL patients to predict response but currently without a recommendation to change therapy in non-responders [19, 20]. Clinical trials concerning the value of a change of the therapeutic regime in non-responding patients based on interim PET, e.g. the PETAL trial (PET-guided therapy of aggressive non-Hodgkin lymphoma) [21, 22] are performed.

The involvement of PI3K, c-MYC and MAPK pathways on glucose uptake, expression of glycolysis-associated genes and cell proliferation in lymphoma is not known yet.

Thus in this study we performed experiments on the regulation of glucose uptake, transcription of genes involved in glycolysis, and regulation of miRNAs by the PI3K/AKT/mTOR pathway, c-MYC and different MAPK signaling pathways. Moreover, we studied the effect of 2-deoxy-d-glucose (2-DG), a glucose analogue that decreases glycolytic rate by inhibiting HK, as a single agent and in combination with inhibitors of the c-MYC-, PI3K-, and MAPK signaling pathway inhibitors on cell viability.

## Methods

### Compounds and chemicals

The c-MYC-Inhibitor 10058-F4 [23], the p38-MAPK inhibitor SB203580 and the GLUT inhibitor phloretin were from Selleck Chemicals (Houston, TX, USA). The PI3K inhibitor LY294002, the mTOR inhibitor Rapamycin and the mitogen-activated protein kinase kinase (MEK) inhibitor PD98059 were from Merck-Millipore (Darmstadt, Germany). The GLUT inhibitor cytochalasin B and CoCl_2_ were from Sigma Aldrich (St. Louis, MO, USA). They were stored in 10 mM aliquots in DMSO at −20 °C and further diluted in the appropriate medium. 2-deoxy-d-glucose (2-DG) was from Sigma-Aldrich and was dissolved in water and stored as 1 M aliquots at −20 °C.

### Cell lines and cell culture

Established human lymphoma cell lines BJAB, OCI-LY3 and SU-DHL-6 were used in this study. BJAB cells were derived from a Burkitt lymphoma, SU-DHL-6 cells from a follicular B cell lymphoma and OCI-LY3 cells from a DLBCL. The BJAB and SU-DHL-6 cell lines were classified as germinal center B cell like (GCB), OCI-LY3 cells were classified as activated B cell like (ABC) [24]. Cell lines were purchased from ATCC (Manassas, VA, USA) and DSMZ (Braunschweig, Germany). Cell lines were maintained in their appropriate media supplemented with 10% fetal bovine serum (FBS, Thermo Fisher, Waltham, MA, USA) at 37 °C at 5% CO_2_.

### FDG uptake studies and protein determination

3 × 10^5^ cells were seeded into each well of a 24 well plate in their appropriate medium containing 0.5% FBS and the inhibitors and concentrations indicated. After incubation for 24 h, cells were harvested by centrifugation and resuspended in glucose-free RPMI medium (Thermo Fisher) supplemented with 5.5 mM glucose [25] and 0.5% FBS and the inhibitors indicated. After 30 min, 100 kBq of FDG was added to the cells and samples were incubated for the indicated times or for 30 min at 37 °C. The cell suspension was pipetted onto a Costar SpinX centrifuge tube filter column (0.45 µm, Corning, Corning, NY, USA) and centrifuged for 1 min at 4 °C to retain the cells with the incorporated radioactivity. The flow through was discarded and cells were washed two times with cold PBS. Cell-bound radioactivity was measured with a gamma counter (2480 Automatic Gamma Counter Wizard^2^ 3, Perkin Elmer, Waltham, MA, USA). A parallel plate was treated in the same way and cells were harvested and lysed in cell lysis buffer (Cell Signaling Technology, Danvers, MA, USA) for. The lysates were centrifuged at 10,000×*g* for 10 min at 4 °C and the protein concentrations of supernatants were determined with a modified Bradford assay (Bio-Rad Laboratories, Hercules, CA, USA).

### RNA and micro RNA isolation and RT-PCR

RNA and miRNA were isolated from the same sample using the RNeasy MinElute Cleanup Kit and the miRNA Kit (Qiagen, Hilden, Germany). In brief, 2 × 10^6^ cells were seeded in each well of a six well plate and incubated with the inhibitors and concentrations indicated for 24 h. After centrifugation (300×*g*, 5 min), the cells were lysed with Qiazol lysis reagent (Qiagen) and RNA >200 nucleotides and small RNAs were isolated separately according to the instructions of the manufacturer.

700 ng of RNA were reverse transcribed in a 20 µl reaction using the QuantiTect RT Kit (Qiagen). For reverse transcription of miRNA, the equivalent volume to 2 µg RNA was incubated with specific primers (Thermo Fisher) and transcribed with the TaqMan miRNA RT-Kit (Thermo Fisher) in a 15 µl reaction. PCR analyses of reversed transcribed RNA and miRNA were performed using 2 µl of the RT reaction in a 20 µl PCR reaction with the 2x TaqMan Universal Master Mix (Thermo Fisher) and TaqMan Gene Expression Assays or TaqMan MicroRNA Assays (Thermo Fisher) on a Step One Plus real time PCR system (Thermo Fisher). For identification of a suitable reference gene, TaqMan array plates with 47 endogenous control gene candidates (Thermo Fisher) were used on the same instrumentation. Amplifications were performed with the following temperature profile: 5 min initial denaturation at 94 °C, followed 45 cycles of denaturation at 94 °C for 30 s and combined primer annealing and extension at 60 °C for 1 min. Fluorescence intensity of FAM was automatically determined during PCR, with ROX as the passive reference. All experiments were performed at least three times and all samples were amplified in triplicate. CT (cycle threshold) values were calculated with the Step One Plus software tool (Thermo Fisher). The ΔΔCT method [26] was used to calculate changes of cDNA levels compared to untreated control and corrected to a reference gene. Excel 2010 (Microsoft, Redmont, WA, USA) was used to calculate mean CT values of the untreated and treated samples ±standard deviations (s_1_ and s_2_), the ΔCT value ± standard deviation (s = s_1_^2^ + s_2_^2^)^1/2^ and the ΔΔCT value ± standard deviation. Statistical differences between two groups were assessed with the Mann–Whitney test using SPSS (IBM Inc, Armonk, NY, USA); p < 0.05 were considered statistically significant.

### Cell survival studies

For cell survival studies, 5 × 10^4^ cells were seeded into each well of 96-well plates in culture medium with 0.5% FBS containing the indicated concentrations of inhibitors or a combination of two substances as indicated. After 48 h, viable cells were stained with the Cell Titer Aqueous One Solution assay (Promega, Madison, WI, USA). Optical density at 490 nm was measured with an Emax microplate photometer (Molecular Devices, Sunnyvale, CA, USA). Control values without treatment were performed as 22-fold determinations, while all concentrations of inhibitors were tested in eightfold. Calculation of results and Student’s t-test were performed using SoftMax pro software (Molecular Devices), and IC50 values were calculated using Sigma Plot software (Systat, San Jose, CA, USA). Interaction of 2-DG with inhibitors was calculated according to the method of Drewinko et al. [27] using the following formula: Cl = (survival a × survival b)/(survival (a + b) * 100), where a and b indicate the two drugs used and a + b indicates the combination of a and b. The interaction was interpreted on the basis of Cl values where Cl > 1.05 indicates synergism, 0.95 ≤ Cl ≤ 1.05 indicates additivity and Cl < 0.95 indicates antagonism.

## Results

### FDG uptake in lymphoma cell lines

**Fig. 1:**
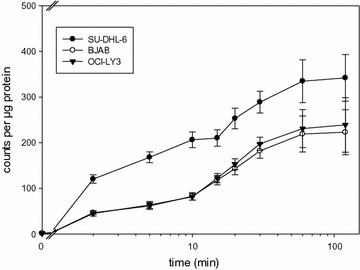
Time-dependent FDG uptake in BJAB, OCI-LY3 and SU-DHL-6 lymphoma cells. Cells were incubated with 100 kBq of FDG for the indicated times and washed as described. Cell-bound radioactivity was normalized to protein concentration determined from a parallel sample. Values represent mean ± standard deviation from fourfold determinations

To characterize the kinetics of FDG uptake in the cells we performed experiments with increasing durations of incubation from 2 min up to 2 h. Results of all three cell lines are shown in Fig. 1. We found that for up to 30 min, FDG uptake, expressed as counts per µg total protein, increased strongly in all three cell lines. After 30 min, uptake increased up to 60 and 120 min, but with a much lower rate (Fig. 1). We therefore decided to perform further experiments with an FDG incubation time of 30 min. FDG uptake per µg protein was comparable in BJAB and OCI-LY3 cells (182.1 and 197.5 counts per µg protein after 30 min of incubation), while SU-DHL-6 cells showed a significantly higher uptake (p < 0.05; 288.7 counts per µg protein; Fig. 1). The reasons for the higher FDG uptake per µg protein in SU-DHL-6 is not known but may be due to the smaller size of SU-DHL-6 cells or to different expression of GLUTs.

**Fig. 2:**
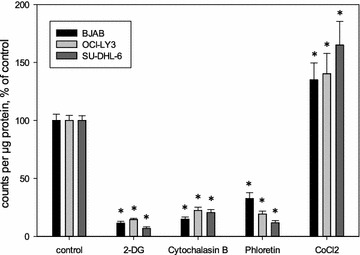
Specificity of FDG uptake in BJAB, OCI-LY3 and SU-DHL-6 cells. Cells were incubated without inhibitors or with 2 mM 2-DG, 5 µM cytochalasin B or 10 µM phloretin for 24 h or with 50 µM CoCl_2_ for 30 min and 100 kBq of FDG for 30 min. Cell-bound radioactivity was normalized to protein concentration determined from a parallel sample and expressed as % of untreated control, mean ± standard deviation from fourfold determinations; *Asterisks* indicates significant decrease or increase (p < 0.05, Student’s t test)

To evaluate the specificity of FDG uptake experiments, we next determined the effect of the GLUT1 inhibitors cytochalasin B and phloretin as well as the glycolysis inhibitor 2-DG and, as a positive control, CoCl_2_ which induces GLUT1 expression (Fig. 2). CoCl_2_ was found to be toxic to the cells and thus used only for 30 min before FDG was added. FDG uptake in cells treated with GLUT1 inhibitors was significantly lower than in untreated cells in all three cell lines indicating the important role of GLUT1 transporter in FDG uptake in the lymphoma cells. Incubation with CoCl_2_ for 30 min significantly increased FDG uptake in all three lymphoma cell lines as expected (Fig. 2). Incubation with 2-DG as a competitor of FDG significantly decreased uptake of FDG as expected (Fig. 2). Taken together, these experiments confirmed the specificity of FDG uptake in our lymphoma cells.

**Fig. 3:**
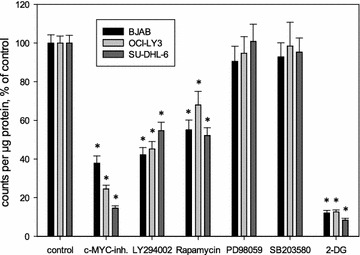
Decreased FDG uptake in BJAB, OCI-LY3 and SU-DHL-6 cells after incubation with c-MYC-inhibitor (5 µM), LY294002 (5 µM) and Rapamycin (500 nM) but not after incubation with PD98059 (10 µM) and SB203580 (10 µM). Cells were incubated without inhibitors or with the inhibitors indicated for 24 h and 100 kBq of FDG was added for 30 min. Cell-bound radioactivity was normalized to protein concentration determined from a parallel sample. Results are expressed as % of untreated control, mean ± standard deviation from fourfold determinations; *Asterisks* indicates significant decrease (p < 0.05, Student’s t test). Results of an incubation with 2-DG (2 mM) are shown as control

The effect of an inhibition of c-MYC (c-MYC inhibitor 10058-F4), PI3K (LY294002), mTOR (Rapamycin), p38-MAPK (SB203580) and MEK (PD98059) was investigated by incubation of the three cell lines with specific inhibitors of these signaling pathways. Concentrations were chosen approx. half the concentrations of IC50 values in proliferation assays (Table 3). While incubation with inhibitors of MAPK (SB203580 as an inhibitor of p38 MAPK and PD98059 as an inhibitor of MEK) had no significant effect on FDG uptake in all three lymphoma cell lines, inhibition of c-MYC (10058-F4), PI3K (LY294002) and mTOR (Rapamycin) led to a significant decrease in FDG uptake (Fig. 3). For the c-MYC inhibitor, the lowest effect with 37.8% of control was observed in BJAB cells, while SU-DHL-6 cells exhibited the most distinct effect (14.6% of control) and FDG uptake in OCI-LY3 cell was modestly decreased (24.6% of control; Fig. 3). LY294002 and Rapamycin also led to a significant decrease of FDG uptake in all three cell lines (Fig. 3). In contrast to c-MYC inhibition, the effect of LY294002 and Rapamycin was most distinct in BJAB cells (42.1 and 55.1% of control) while OCI-LY3 and SU-DHL-6 cells showed a significant decrease compared to untreated controls, but to a lesser extent than BJAB cells (LY294002: OCI-LY3 cells 45.2%; SU-DHL-6 cells 54.7% of control; Rapamycin: OCI-LY3 cells 68.0%; SU-DHL-6 cells 52.2% of control; Fig. 3). As a control, 2-DG led to a decrease of FDG uptake to values around 10% of control in all three cell lines (Fig. 3).

### PCR analyses

In order to identify a reference gene that is not regulated by our experimental treatment and thus suitable for normalization in real-time PCR assays, we used TaqMan array plates each with 47 endogenous control gene candidates. cDNA from untreated BJAB cells as well as cDNA from BJAB cells treated with the c-MYC inhibitor, LY294002 and Rapamycin was analyzed using the array plates. The hypoxanthine phosphoribosyltransferase 1 (HPRT1) gene showed the lowest regulation of all endogenous control gene candidates analyzed (less than 5% up and downregulation after each of the three treatments compared to untreated control) and was chosen as the most suitable reference gene for the cDNA measurements. For SB203580 and PD294002 treatments as well as in OCI-LY3 and SU-DHL-6 cells, the suitability of the HPRT1 gene as reference gene was verified within the following PCR analyses. All deviations compared to untreated controls were <8%.

For miRNA normalization, six candidate miRNAs (U6, RNU48, miR16, miR24, miR26a and miR28-5p) were selected that were shown to be not regulated in other cell models [28, 29]. All six miRNAs were analyzed within the PCR assays. U6 was found to be the miRNA least regulated (<8% compared to control in all three cell lines analyzed and all treatments).

To ensure similar PCR efficiencies as a prerequisite for relative quantification with the ΔΔCT method, PCR efficiency of each primer and probe set was first determined by measuring serial tenfold dilutions of two templates. PCR efficiency was calculated according to the equation: PCR efficacy = (10 ^[−1/slope]^ −1) * 100 [30]. All PCR assays had an efficiency of >95% (96–101%) as described by the manufacturer.

We first analyzed mRNA expression of the GLUT1 gene after incubation with the five inhibitors. Incubation with the c-MYC inhibitor and the mTOR inhibitor Rapamycin after 24 h led to a significant down-regulation of GLUT1 mRNA in all three cell lines (Table 1). LY294002 did not affect expression of GLUT1 mRNA (Table 1). Incubation with the MEK inhibitor PD98059 and the p38 MAPK inhibitor SB203580 did not change expression of the GLUT1 mRNAs significantly (Table 1).

**Table 1:** Changes of mRNA expression in BJAB, OCI-LY3 and SU-DHL-6 cells after incubation with the c-MYC-inhibitor (5 µM), LY294002 (5 µM), Rapamycin (500 nM), PD98059 (10 µM) and SB203580 (10 µM) for 24 h

	GLUT1	HK2	G6Ptase	LDHA
BJAB cells				
*c-MYC-inh.*				
ΔΔCT	1.71 ± 0.11 ↓	−0.45 ± 0.16 ↑	−1.69 ± 0.14 ↑	1.09 ± 0.14 ↓
Fold of untreated	0.31 (0.28–0.33)*	1.37 (1.22–1.53)*	3.23 (2.93–3.56)*	0.47 (0.43–0.52)*
*LY294002*				
ΔΔCT	0.03 ± 0.10	0.08 ± 0.09	−0.41 ± 0.10 ↑	1.24 ± 0.17 ↓
Fold of untreated	0.98 (0.91–1.06)	0.95 (0.89–1.01)	1.33 (1.24–1.42)*	0.42 (0.38–0.48)*
*Rapamycin*				
ΔΔCT	0.78 ± 0.09 ↓	0.84 ± 0.08 ↓	−0.72 ± 0.17 ↑	1.33 ± 0.14 ↓
Fold of untreated	0.58 (0.55–0.62)*	0.56 (0.53–0.59)*	1.65 (1.46–1.85)*	0.40 (0.36–0.44)*
*PD98059*				
ΔΔCT	−0.02 ± 0.13	0.01 ± 0.10	−0.45 ± 0.09 ↑	0.04 ± 0.10
Fold of untreated	1.01 (0.93–1.11)	0.99 (0.93–1.06)	1.37 (1.28–1.45)*	0.97 (0.91–1.04)
*SB203580*				
ΔΔCT	0.01 ± 0.08	−0.02 ± 0.07	0.05 ± 0.11	−0.09 ± 0.12
Fold of untreated	0.99 (0.94–1.05)	1.01 (0.97–1.06)	0.97 (0.90–1.04)	1.06 (0.98–1.16)
OCI-LY3 cells				
*c-MYC-inh.*				
ΔΔCT	1.12 ± 0.14 ↓	−0.43 ± 0.12 ↑	−1.23 ± 0.17 ↑	0.78 ± 0.12 ↓
Fold of untreated	0.46 (0.42–0.51)*	1.35 (1.24–1.46)*	2.35 (2.08–2.64)*	0.58 (0.54–0.63)*
*LY294002*				
ΔΔCT	0.13 ± 0.15	−0.04 ± 0.10	−0.60 ± 0.10 ↑	1.03 ± 0.14 ↓
Fold of untreated	0.91 (0.82–1.01)	1.03 (0.96–1.10)	1.52 (1.41–1.62)*	0.49 (0.44–0.54)*
*Rapamycin*				
ΔΔCT	0.49 ± 0.13 ↓	0.98 ± 0.15 ↓	−0.97 ± 0.10 ↑	1.56 ± 0.20 ↓
Fold of untreated	0.71 (0.65–0.78)*	0.51 (0.46–0.56)*	1.96 (1.83–2.10)*	0.34 (0.30–0.39)*
*PD98059*				
ΔΔCT	−0.03 ± 0.18	0.01 ± 0.13	−0.03 ± 0.08	0.01 ± 0.16
Fold of untreated	1.02 (0.90–1.16)	0.99 (0.91–1.09)	1.02 (0.97–1.08)	0.99 (0.89–1.11)
*SB203580*				
ΔΔCT	0.16 ± 0.14	0.44 ± 0.12 ↓	−0.08 ± 0.13	−0.03 ± 0.08
Fold of untreated	0.90 (0.81–0.99)	0.74 (0.68–0.80)*	1.06 (0.97–1.16)	1.02 (0.97–1.08)
SU-DHL-6 cells				
*c-MYC-inh.*				
ΔΔCT	1.23 ± 0.12 ↓	−0.36 ± 0.07 ↑	−0.93 ± 0.12 ↑	1.19 ± 0.08 ↓
Fold of untreated	0.43 (0.39–0.46)*	1.28 (1.22–1.35)*	1.91 (1.75–2.07)*	0.44 (0.41–0.46)*
*LY294002*				
ΔΔCT	−0.11 ± 0.11	−0.05 ± 0.14	0.51 ± 0.08 ↑	0.98 ± 0.11 ↓
Fold of untreated	1.08 (1.00–1.16)	1.04 (0.94–1.14)	1.42 (1.35–1.51)*	0.51 (0.47–0.55)*
*Rapamycin*				
ΔΔCT	0.66 ± 0.09 ↓	−0.83 ± 0.16 ↓	−0.82 ± 0.16 ↑	0.85 ± 0.09 ↓
Fold of untreated	0.63 (0.59–0.67)*	0.56 (0.50–0.63)*	1.77 (1.58–1.97)*	0.55 (0.52–0.59)*
*PD98059*				
ΔΔCT	0.04 ± 0.16	0.09 ± 0.11	−0.05 ± 0.13	0.30 ± 0.10 ↓
Fold of untreated	0.97 (0.87–1.09)	0.94 (0.87–1.01)	1.04 (0.95–1.13)	0.81 (0.76–0.87)*
*SB203580*				
ΔΔCT	−0.08 ± 0.15	0.05 ± 0.09	0.14 ± 0.14	0.17 ± 0.17
Fold of untreated	1.06 (0.95–1.17)	0.97 (0.91–1.03)	0.91 (0.82–1.00)	0.89 (0.79–1.00)

ΔΔCT values referred to changes of mRNA levels corrected to expression of HPRT1 mRNA; changes of expression values represent percent of control, mean ± standard deviation from threefold determinations

* and arrows indicate significant changes (p < 0.05, Mann–Whitney test)

Treatment with the c-MYC inhibitor in all three cell lines increased expression of HK2 mRNA (Table 1). After incubation with Rapamycin, expression of HK2 mRNA was decreased in all three cell lines, while LY294002 had no significant effect on HK2 mRNA (Table 1). Incubation PD98059 and SB203580 did not change expression of HK2 mRNA significantly (Table 1).

Lactate dehydrogenase A (LDHA) expression was significantly decreased by the c-MYC inhibitor, Rapamycin and LY294002 in all three cell lines (Table 1). In SU-DHL-6 cells PD98059 incubation led to a slight, but significant decrease in LDHA mRNA expression, while expression of LDHA was not significantly affected by PD98059 in the other cells. Treatment with SB 203580 did not affect mRNA levels of LDHA in all three cell lines (Table 1).

Glucose-6-phosphatase (G6Pase) expression was found to be positively regulated by the c-MYC inhibitor, Rapamycin and LY294002 in all three cell lines. PD98059 incubation led to an increase only in BJAB cells, while G6Pase mRNA remained unchanged after this treatment in SU-DHL-6 and OCI-LY3 cells. SB203580 incubation did not affect G6Pase mRNA expression in all three cell lines (Table 1).

miRNA21 expression was diminished by the c-MYC inhibitor and Rapamycin in all three cell lines, while LY294002 incubation led to a decrease in miRNA21 only in BJAB and OCI-LY3 cells (Table 2). miRNA21 expression was not significantly influenced by treatment with PD98059 and SB203580 (Table 2).

**Table 2:** Changes of miRNA expression in BJAB, OCI-LY3 and SU-DHL-6 cells after incubation with the c-MYC-inhibitor (5 µM), LY294002 (5 µM), Rapamycin (500 nM), PD98059 (10 µM) and SB203580 (10 µM) for 24 h

	miR21	miR23a	miR133a	miR133b	miR138-1	miR143
BJAB cells						
*c-MYC-inh.*						
ΔΔCT	1.78 ± 0.13 ↓	0.16 ± 0.11	0.80 ± 0.14 ↓	−0.89 ± 0.14 ↑	−1.68 ± 0.17 ↑	−0.12 ± 0.14
Fold of untreated	0.29 (0.27–0.32)*	0.90 (0.83–0.97)	0.57 (0.54–0.61)*	1.85 (1.68–2.04)*	3.20 (2.85–3.61)*	1.09 (0.99–1.20)
*LY294002*						
ΔΔCT	1.06 ± 0.18 ↓	0.21 ± 0.19	1.02 ± 0.20 ↓	0.52 ± 0.12 ↑	−0.54 ± 0.09 ↑	0.06 ± 0.11
Fold of untreated	0.48 (0.42–0.54)*	0.86 (0.76–0.99)	0.49 (0.43–0.57)*	1.43 (1.32–1.56)*	1.45 (1.37–1.55)*	0.96 (0.89–1.04)
*Rapamycin*						
ΔΔCT	1.28 ± 0.08 ↓	0.27 ± 0.24	−0.64 ± 0.15 ↑	−0.86 ± 0.12 ↑	−0.53 ± 0.11 ↑	0.21 ± 0.20
Fold of untreated	0.41 (0.39–0.44)*	0.83 (0.70–0.98)	1.56 (1.40–1.73)*	1.82 (1.67–1.97)*	1.44 (1.34–1.56)*	0.86 (0.75–0.99)
*PD98059*						
ΔΔCT	0.03 ± 0.08	−0.04 ± 0.07	−0.65 ± 0.12 ↑	−0.60 ± 0.15 ↑	−1.51 ± 0.14 ↑	0.05 ± 0.10
Fold of untreated	0.98 (0.93–1.04)	1.03 (0.98–1.08)	1.57 (1.44–1.71)*	1.52 (1.37–1.68)*	2.85 (2.58–3.14)*	0.97 (0.90–1.04)
*SB203580*						
ΔΔCT	−0.04 ± 0.13	−0.01 ± 0.11	0.03 ± 0.07	−0.01 ± 0.07	−0.05 ± 0.12	0.06 ± 0.09
Fold of untreated	1.03 (0.94–1.13)	1.01 (0.93–1.09)	0.98 (0.93–1.03)	1.01 (0.96–1.06)	1.04 (0.95–1.13)	0.96 (0.90–1.02)
OCI-LY3 cells						
*c-MYC-inh.*						
ΔΔCT	1.20 ± 0.15 ↓	0.19 ± 0.17	0.83 ± 0.12 ↓	−0.70 ± 0.13 ↑	−1.47 ± 0.16 ↑	−0.06 ± 0.11
Fold of untreated	0.44 (0.39–0.48)*	0.88 (0.78–0.99)	0.56 (0.52–0.61)*	1.62 (1.48–1.78)*	2.77 (2.48–3.10)*	1.04 (0.97–1.13)
*LY294002*						
ΔΔCT	0.76 ± 0.14 ↓	0.05 ± 0.09	0.61 ± 0.14 ↓	−0.77 ± 0.13 ↑	−0.84 ± 0.18 ↑	0.13 ± 0.12
Fold of untreated	0.59 (0.54–0.65)*	0.97 (0.91–1.03)	0.66 (0.59–0.72)*	1.71 (1.56–1.87)*	1.79 (1.58–2.03)*	0.91 (0.84–0.99)
*Rapamycin*						
ΔΔCT	1.45 ± 0.17 ↓	0.20 ± 0.18	−0.80 ± 0.16 ↑	−0.60 ± 0.10 ↑	−0.93 ± 0.12 ↑	−0.02 ± 0.06
Fold of untreated	0.37 (0.33–0.41)*	0.87 (0.77–0.99)	1.74 (1.56–1.95)*	1.52 (1.40–1.64)*	1.91 (1.75–2.07)*	1.01 (0.97–1.06)
*PD98059*						
ΔΔCT	−0.04 ± 0.08	0.26 ± 0.15	−0.77 ± 0.13 ↑	−0.53 ± 0.13 ↑	0.06 ± 0.10	−0.14 ± 0.16
Fold of untreated	1.03 (0.97–1.09)	0.90 (0.81–0.99)	1.71 (1.56–1.87)*	1.44 (1.32–1.58)*	0.96 (0.90–1.03)	1.10 (0.99–1.23)
*SB203580*						
ΔΔCT	0.05 ± 0.08	−0.05 ± 0.07	−0.17 ± 0.15	0.09 ± 0.13	0.00 ± 0.09	−0.14 ± 0.13
Fold of untreated	0.97 (0.91–1.02)	1.04 (0.99–1.09)	0.89 (0.80–0.99)	0.94 (0.86–1.03)	1.00 (0.94–1.06)	1.10 (1.01–1.21)
SU-DHL-6 cells						
*c-MYC-inh.*						
ΔΔCT	0.68 ± 0.13 ↓	−0.03 ± 0.07	ne	−0.99 ± 0.17 ↑	0.08 ± 0.11	ne
Fold of untreated	0.62 (0.57–0.68)*	1.02 (0.97–1.07)		1.99 (1.77–2.23)*	0.95 (0.81–1.02)	
*LY294002*						
ΔΔCT	0.12 ± 0.20	−0.21 ± 0.24	ne	0.73 ± 0.10 ↓	−0.03 ± 0.13	ne
Fold of untreated	0.92 (0.80–1.06)	1.16 (0.98–1.37)		0.60 (0.56–0.65)*	1.02 (0.93–1.12)	
*Rapamycin*						
ΔΔCT	1.14 ± 0.14 ↓	0.05 ± 0.07	ne	−0.77 ± 0.13 ↑	−0.07 ± 0.11	ne
Fold of untreated	0.45 (0.41–0.50)*	0.97 (0.92–1.01)		1.71 (1.56–1.87)*	1.05 (0.97–1.13)	
*PD98059*						
ΔΔCT	−0.08 ± 0.14	0.06 ± 0.07	ne	−0.07 ± 0.11	−0.01 ± 0.06	ne
Fold of untreated	1.06 (0.96–1.16)	0.96 (0.91–1.01)		1.05 (0.97–1.13)	1.01 (0.97–1.05)	
*SB203580*						
ΔΔCT	−0.10 ± 0.09	−0.06 ± 0.10	ne	0.08 ± 0.08	0.03 ± 0.12	ne
Fold of untreated	1.07 (1.01–1.14)	1.04 (0.97–1.12)		0.95 (0.90–1.00)	0.98 (0.90–1.06)	

ΔΔCT values referred to changes of miRNA levels corrected to expression of U6 miRNA; changes of expression values represent percent of control, mean ± standard deviation from threefold determinations

* and arrows indicate significant changes (p < 0.05, Mann–Whitney test)

*ne* not expressed

Expression of miRNA23a was not significantly influenced by the five inhibitors used here. miRNA133a was diminished by the c-MYC inhibitor and LY294002 and increased by Rapamycin and PD98059 in BJAB and OCI-LY3 cells, while in SU-DHL-6 cells miRNA133a expression was increased by the c-MYC inhibitor and not affected by the other inhibitors (Table 2).

For miRNA133b-, -138-1- and -143 expression, SU-DHL-6 cells also showed an expression pattern distinct from that in BJAB and OCI-LY3 cells: miRNA133b was increased after treatment with the c-MYC inhibitor, Rapamycin, LY294002 and PD98059, while SB203580 had no significant effect in BJAB and OCI-LY3 cells. In SU-DHL-6 cells, only the c-MYC inhibitor and Rapamycin led to a significant increase in miRNA133b, while LY294002 treatment decreased miRNA133b expression and PD98059 and SB203580 incubation had no effect on miRNA133b (Table 2).

miRNA138-1 expression was not affected by all five inhibitors in SU-DHL-6 cells, while in BJAB and OCI-LY3 cells treatment with the c-MYC inhibitor, Rapamycin and LY294002 resulted in an increased expression of miRNA138-1. PD98059 also increased miRNA138-1 in BJAB cells but not in OCI-LY3 cells, while incubation with SB203580 had no significant effect (Table 2).

miRNA143 was not expressed in SU-DHL-6 cells. In BJAB and OCI-LY3 cells, miRNA143 showed no regulation after incubation with all five inhibitors (Table 2).

miRNA195-5p was regulated non-uniformly in the three cell lines: in BJAB cells it was upregulated by LY294002, in OCI-LY3 cells it was upregulated by PD98059 and SB203580 and downregulated by LY294002, while in SU-DHL-6 cells it was downregulated by the c-MYC inhibitor (Table 2).

### The c-MYC inhibitor, LY294002, Rapamycin and 2-DG decreased viability of lymphoma cells

**Fig. 4:**
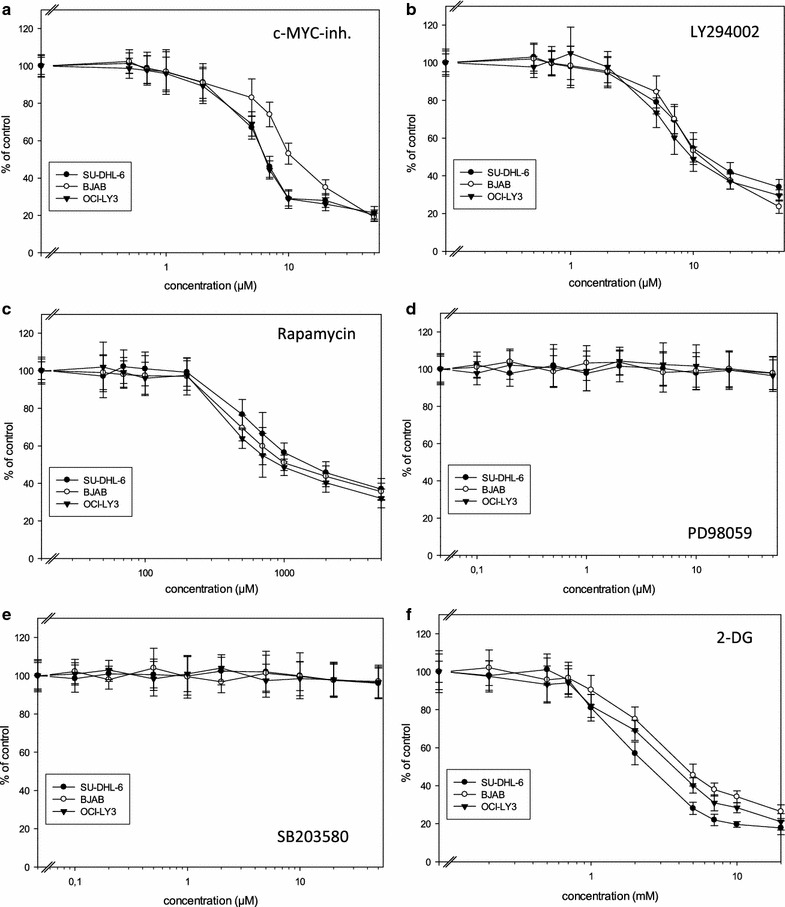
Decreased viability of BJAB, OCI-LY3 and SU-DHL-6 cells after incubation with the c-MYC-inhibitor (**a**), LY294002 (**b**), Rapamycin (**c**) and 2-DG (**f**) but not after incubation with PD98059 (**d**) and SB203580 (**e**). Cells were cultured in the presence of increasing concentrations of the substances indicated or vehicle control for 48 h and viability was assessed by MTT assay. Values represent percent of vehicle control, mean ± standard deviation from eightfold determinations

The three lymphoma cell lines were treated with increasing concentrations of the c-MYC inhibitor, LY294002, Rapamycin, SB203580, PD98059 as well as with 2-DG or vehicle for 48 h and the percentage of viable cells compared to controls was assessed. Treatment with SB203580 and PD98059 with concentrations up to 50 µM had no significant effect on survival of all three cell lines (Fig. 4d, e; Table 3). In contrast, incubation with the c-MYC inhibitor decreased the number of viable cells in all cell lines examined with OCI-LY3 being the most sensitive cell line (IC50 values: 10.23 µM for BJAB; 6.48 µm for OCI-LY3 and 6.67 µM for SU-DHL-6; Fig. 4a; Table 3). LY294002 also significantly decreased the number of viable cells in all three cell lines with IC50 values in the same range in the three cell lines (BJAB: 10.66 µM; OCI-LY3: 9.67 µM; SU-DHL-6: 10.94 µM; Fig. 4b; Table 3). Rapamycin was also effective with similar IC50 values in all three lymphoma cell lines (BJAB: 1021 nM; OCI-LY3: 912 nM; SU-DHL-6: 1208 nM; Fig. 4c; Table 3).

**Table 3:** IC50 values of BJAB, OCI-LY3 and SU-DHL-6 cells after 48 h of treatment with increasing concentrations of the c-MYC-inhibitor, LY294002, Rapamycin, PD98059, SB203580 and 2-DG (MTT assay)

	BJAB	OCI-LY3	SU-DHL-6
c-MYC-inh (µM)	10.23	6.48	6.67
LY294002 (µM)	10.66	9.67	10.94
Rapamycin (nM)	1021	912	1208
PD98059 (µM)	>50	>50	>50
SB203580 (µM)	>50	>50	>50
2-DG (mM)	4.65	4.07	2.86

Cell viability of all three cell lines was inhibited after incubation with 2-DG, an inhibitor of hexokinase, with the SU-DHL-6 cell line being the most sensitive cell line (IC50: BJAB: 4.65 mM; OCI-LY3: 4.073 mM; SU-DHL-6: 2.86 mM; Fig. 4f; Table 3).

### Synergistic action of 2-DG with inhibitors

The effects of a combined treatment of the three lymphoma cell lines with 2-DG and the signaling pathways inhibitors on the survival rates were investigated to study the possibility of a synergistic effect. The results are shown in Table 4. Calculation of survival rates according to the method of Drewinko et al. [27] yielded synergistic effects (Cl values >1.05) in all three cell lines for the combination of 2-DG with the c-MYC-inhibitor 10058-F4 and with LY294002 (Table 4a, b), while for the combination of 2-DG with Rapamycin the BJAB and SU-DHL-6 cells showed synergistic effects and OCI-LY3 cells had an additive effect (Table 4c). Interestingly, we observed also synergistic effects of 2-DG with the p38 MAPK inhibitor SB203580 in all three cell lines (Table 4e), though SB203580 alone had no effects on viability of cells (Fig. 4d). The combination of 2-DG and PD98059 was synergistic in OCI-LY3 cells and had additive effects in BJAB and SU-DHL-6 cells (Table 4d). No antagonistic effect was observed (Table 4).

**Table 4:** Interaction of 2-DG (1 mM) with c-MYC-inhibitor (2 µM; a), LY294002 (5 µM; b), Rapamycin (200 nM; c), PD98059 (5 µM; d) and SB203580 (5 µM; e) in the three lymphoma cell lines

a cell line	2-DG 1 mM	c-MYC-inh. 2 µM	2-DG + c-MYC-inh.	CI
BJAB	89.6 ± 7.4	91.0 ± 7.7	57.7 ± 4.8	1.41 syn.
OCI-LY3	81.0 ± 6.6	85.4 ± 7.3	56.3 ± 5.5	1.23 syn.
SU-DHL-6	84.0 ± 6.3	86.3 ± 6.4	57.9 ± 5.1	1.25 syn.

b cell line	2-DG 1 mM	LY294002 5 µM	2-DG + Y294002	CI
BJAB	88,1 ± 7.0	84.6 ± 7.0	47.2 ± 3.5	1.60 syn.
OCI-LY3	79.0 ± 7.3	73.7 ± 6.6	42.6 ± 4.6	1.37 syn.
SU-DHL-6	83.5 ± 6.4	87.9 ± 8.1	38.2 ± 3.1	1.92 syn.

c cell line	2-DG 1 mM	Rapamycin 200 nM	2-DG + Rapamycin	CI
BJAB	90.7 ± 6.8	90.4 ± 8.3	58.2 ± 3.7	1.41 syn.
OCI-LY3	80.5 ± 6.4	88.9 ± 7.6	69.5 ± 5.0	1.03 add.
SU-DHL-6	82.5 ± 7.6	91.6 ± 7.9	47.8 ± 3.4	1.58 syn

d cell line	2-DG 1 mM	PD98059 5 µM	2-DG + PD98059	CI
BJAB	90.3 ± 8.4	103.7 ± 9.2	95.7 ± 7.7	0.98 add.
OCI-LY3	81.5 ± 6.8	98.2 ± 8.5	75.3 ± 5.6	1.06 syn.
SU-DHL-6	81.5 ± 5.8	99.4 ± 7.8	77.9 ± 6.5	1.04 add.

e cell line	2-DG 1 mM	SB203580 5 µM	2-DG + SB203580	CI
BJAB	91.4 ± 6.7	102.6 ± 9.0	83.5 ± 7.0	1.12 syn.
OCI-LY3	80.8 ± 7.4	97.8 ± 7.6	63,5 ± 5.3	1.24 syn.
SU-DHL-6	79.6 ± 5.7	104.3 ± 8.4	54.1 ± 4.5	1.53 syn.

MTT assays were performed to determine the viability of cells after incubation with one compound alone or in combination. Cl values were calculated according to the method of Drewinko et al. (see “Methods”), where Cl > 1.05 indicates synergism (syn.), 0.95 ≤ Cl ≤ 1.05 indicates additivity (add.) and Cl < 0.95 indicates antagonism

## Discussion

In this study we showed regulation of glucose uptake by c-MYC- and PI3K/AKT/mTOR dependent pathways in lymphoma cell lines and demonstrated evidence for the altered glucose metabolism as a potential target to improve inhibitor-based therapeutic approaches in these cells.

During tumorigenesis, a change in energy metabolism of the tumor cells occurs and deregulated metabolism of glucose is one hallmark of cancer [31]. The exact molecular mechanisms leading to this altered phenotype of cancer cells remain unclear. Besides hypoxia, activation of oncogenes and inactivation of tumor suppressor genes, alterations in the cellular signaling network are involved in the glycolytic switch in cancer cells [Review: 32].

### Effects of PI3K/mTOR inhibitors

The PI3K/AKT/mTOR pathway is altered in many human cancers by activating mutations, aberrant receptor tyrosine kinase signaling or inactivating mutations in tumor suppressor genes like PTEN (phosphatase and tensin homolog) [33, 34]. In NHL, this pathway is often activated, although mutations are only infrequently found [Review: 35]. AKT as a downstream effector of PI3K is known as an important driver of the tumor glycolytic phenotype and renders cancer cells dependent on glycolysis for survival [9, 36]. AKT stimulates glucose uptake and glycolysis by increasing the expression and membrane translocation of glucose transporter proteins like GLUT1 and by activating glycolytic enzymes and regulating HK expression, activity and interaction with mitochondria [37]. mTOR is a serine/threonine kinase downstream of PI3K-AKT that acts through the mTOR complexes 1 and 2 (mTORC1 and -2) to induce transcription of many genes involved in altered tumor cell metabolism [38].

Treatment of our NHL cell lines with the PI3K inhibitor LY294002 or the mTOR inhibitor Rapamycin led to a decrease in cell viability in all three cell lines with IC50 values in the range of around 1 µM for Rapamycin and 10 µM for LY294002 (Fig. 4; Table 1) demonstrating the importance of the PI3K pathway for survival of the cells. These findings are in good agreement with recently reported results of Ezell and coworkers [39] who found PI3K and mTOR inhibitors effective in arresting proliferation in DLBCL lines. However, response rates to mTOR inhibitors as single agent therapy in phase II clinical studies are only around 30% in NHL [40, 41].

In addition to the effects on the number of viable cells, both inhibitors led to a decreased FDG uptake after 24 h treatment as expected (Fig. 3). A similar inhibitory effect of PI3K/mTOR inhibitors on FDG uptake was already described in cervical cancer cells [42] and confirms the well-known importance of the PI3K pathway for enhanced glucose metabolism.

Unexpectedly, while Rapamycin led to a decrease in GLUT1 mRNA expression, treatment with LY294002 did not significantly affect GLUT1 mRNA in all three cell lines (Table 2). This discrepancy between mRNA results and FDG uptake after LY294002 treatment may be due to modifications in protein expression and processing like reduced translocation of GLUT1 and GLUT4 to the cell membrane as described in cervical cancer and lung adenocarcinoma cells [42, 43]. In cutaneous T-cell lymphoma cells, incubation with Rapamycin decreased glucose uptake in both cell lines investigated, but mRNA expression of glycolysis genes was diminished only in one cell line [44]. These results show that the molecular mechanism by which the PI3K signaling cascade regulates GLUT processing is still not clear and further experiments are needed to elucidate the modulation of expression, translocation and regulation of GLUTs.

The decrease of LDHA mRNA expression as well as the increase in G6Ptase after incubation with LY294002 and Rapamycin in all three cell lines corresponds to a diminished glycolysis after PI3K/AKT/mTOR inhibition, since LDHA is a glycolytic enzyme while G6Ptase catalyzes the reaction from glucose-6-phosphate back to glucose [3].

HK2 mRNA expression was diminished in all three cell lines after incubation with Rapamycin, but not with LY294002. This may reflect the distinct levels of inhibition by the two substances. LY294002 inhibits PI3K, while Rapamycin inhibits mTOR proteins that are located downstream of PI3K and AKT. Treatment with LY294002 may therefore be circumvented by activation of the downstream AKT and mTOR kinases by alternative pathways or mutations and thus may explain the different effects of LY294002 and Rapamycin in our cells [45].

The changes in miRNA expression after treatment with LY294002 or Rapamycin primarily reflect the oncogenic features of the PI3K/mTOR pathway in NHL cells with downregulation of the oncogenic miRNA21 and upregulation of the tumor suppressor miRNAs 133a, -133b and -138-1 (Table 3). Further experiments will show if this regulation is directly related to the diminished expression of the GLUT1 and LDHA genes after inhibition of this pathway and if manipulation of the miRNA level may influence expression of glycolysis-related genes.

### Effect of the c-MYC inhibitor

c-MYC is an oncogenic transcription factor that is overexpressed in many cancer types including B- and T-cell malignancies and is involved in cell metabolism and cell proliferation [46]. In lymphoma, c-MYC activation occurs by several molecular mechanisms including translocations, amplification, mutations, altered intracellular localization of the c-MYC protein or miRNA-dependent mechanisms [47–49].

Incubation of our cells with the c-MYC inhibitor as expected led to a decreased number of viable cells (Fig. 4a) with IC50 values (6.48–10.23 µM) in the lower range seen in other cell systems (breast cancer cells: 20–30 µM [50]; multiple myeloma cell lines: 12–45 µM [51]; ovarian carcinoma cell lines: 3.2 and 4.4 µM [52]; HepG2 hepatocellular carcinoma cells: around 100 µM [53]; acute myeloid leukemia (AML) cell lines: 60–90 µM [54]). Thus, the c-MYC inhibitor may be a suitable substance for reducing the number of viable lymphoma cells. Further experiments may reveal the mode of action, e.g. apoptosis induction, inhibition of proliferation and cell cycle arrest as well as its in vivo efficacy in lymphoma.

Besides a reduction of the number of viable cells, treatment of lymphoma cells with the c-MYC inhibitor led to a marked decrease in FDG uptake in all three cell lines with the SU-DHL-6 cells showing the most pronounced effect (Fig. 3). Corresponding to the decreased FDG uptake, after incubation with the c-MYC inhibitor the expression of GLUT1 mRNA was significantly decreased in all three cell lines (Table 2). Similar effects of a decreased glucose uptake and a diminished expression of GLUT 1 after incubation with the c-MYC inhibitor or after knockdown of c-MYC with siRNA were already described in breast cancer cells [50]. Further experiments are necessary to evaluate protein expression and cellular localization of glucose transporters to determine the cellular effect of an inhibition of c-MYC.

As expected, expression of the mRNA encoding LDHA was also diminished after incubation with the c-MYC-inhibitor (Table 2) while expression of HK2 and the G6Pase was enhanced. Targeting of the expression of glycolytic enzymes by c-MYC was already described in other cell systems [55, 56]. However, while we still do not have an explanation for the increased expression of HK2, enhanced G6Pase expression may be explained similarly as the one after inhibition of the PI3K pathway and may reflect a partly reversed Warburg effect.

The changes in miRNA expression after c-MYC inhibition resemble those after inhibition of PI3K/mTOR probably reflecting the function of c-MYC as an oncogene in NHL cells. In contrast to the results obtained after mTOR inhibition with Rapamycin, after treatment with the c-MYC inhibitor or LY294002, the expression of the miRNA133a was diminished (Table 3). Thus, c-MYC is involved in pathways stimulating proliferation and also enhances the glycolytic phenotype of lymphoma cells. Inhibition of c-MYC therefore is an attractive strategy to inhibit both the altered glucose metabolism and proliferation in lymphoma cells. Further experiments are needed to characterize the exact molecular relationship between glycolytic inhibition and cell death and its mechanisms.

### Effect of inhibitors of MAPK pathways

The p38-MAPK signaling pathway is involved in many cellular functions like differentiation, proliferation and induction of cell death. The exact role of p38-MAPK in a cancer cell depends on the cell type and the tumor stage [57]. In NHL, upregulation was shown in DLBCL [58] and an increased level of phosphorylated p38-MAPK has been correlated with malignancy and failure of response to CHOP treatment [59, 60]. In our experiments, we did not find an influence of p38-MAPK inhibition on cell survival and proliferation, FDG uptake and expression of glycolysis-related genes and miRNAs. These results fit those published by Vega et al. [61] who reported on the lack of apoptosis-inducing effects of SB203580 in NHL cells. Furthermore, Elenitoba-Johnson et al. [58] reported that SB203580 had no effect on proliferation in one of three NHL cell lines, while p38-MAPK inhibition was effective in the other two indicating that the effect of a p38-MAPK inhibition is dependent on presently unknown cell line characteristics.

The MEK pathways are activated mainly in response to the stimulation of tyrosine kinase receptors [62]. MEK pathways are shown to be involved in an enhanced glucose uptake and the metabolic shift in cancer types with an activated BRAF-MAPK pathway by BRAF mutations like those found in melanoma [63]. Furthermore, MAPK pathways were shown to interact with enhanced glutamine metabolism in melanoma cells: In BRAF-mutated melanoma cells that were MAPK inhibitor-resistant, a greater uptake of glutamine and an increased sensitivity to glutamine was demonstrated compared to MAPK inhibitor-sensitive cells [64]. In addition, inhibitors of glutaminase were more efficient in MAPK-inhibitor-resistant cells with regard to decreased cell survival indicating that besides glucose metabolism, glutamine metabolism may be a suitable therapeutic target in cancer cells [64]. In lymphoma, no mutational activation of members of the MAPK pathways are described except in pediatric-type nodal follicular lymphoma, a variant of follicular lymphoma with invariably benign behavior, with a mutation frequency in the *MEK1* gene of 43% [65]. The data presented here show no influence of the MEK inhibitor PD98059 on the number of viable cells, glucose uptake and the expression of glycolysis-related genes and thus fit these literature data. Interestingly, we found an upregulation of the tumor suppressor miRNAs133a, -133b and -138-1 after PD98059 treatment indicating the involvement of MAPK in presently unknown oncogenic pathways.

### Effect of the glucose analog 2-DG

The glucose analog 2-DG is an inhibitor of HK that is used to block the Warburg effect in cancer cells [66]. 2-DG is taken up into the cells via GLUTs and phosphorylated by HK to 2-deoxyglucose-6-phosphate which cannot be further metabolized and thus accumulates in the cell and interferes with the glycolytic pathway by inhibiting HK and phosphoglucose isomerase [66, 67].

Due to its ability to inhibit glycolysis, 2-DG has been evaluated as an anticancer agent in several cell systems [Review: 68]. In our lymphoma cell lines we found a decrease in cell viability after incubation with 2-DG for 48 h with IC50 values in the range of 2.86 mM to 4.65 mM with SU-DHL-6 being the most sensitive cell line (Fig. 4f; Table 1). The IC50 values found here are in the lower range of values reported in the literature for other cell systems (MCF7 breast cancer cells: 6.7 mM; LNCaP prostate cancer cells: 8.1 mM [69]).

Although 2-DG decreases the number of viable cells in short-time cell culture experiments, it has not been effective as a single agent in vivo [68]. We therefore combined 2-DG with the inhibitors used in this study and investigated the effects of combined inhibition on cell viability (Table 2). Approx. half the concentrations used in the other experiments were used for combined treatment (Table 2). Synergistic effects were observed with the c-MYC-inhibitor 10058-F4, with LY294002 and with the p38 MAPK inhibitor SB203580 as well as with Rapamycin in 2 of 3 cell lines (Table 2).

A synergistic effect of a combined treatment with 2-DG and PI3K/mTOR inhibitors as found in our experiments has already been described by a few authors: In lung cancer cell lines, an analogue of Rapamycin hypersensitized cells to 2-DG treatment under hypoxic conditions [70]. Furthermore, a dual PI3K/mTOR inhibitor has recently been reported to have synergistic effect with 2-DG on cell survival in two cell lines of primary effusion lymphoma (PEL), a rare subtype of B-cell NHL [71]. A possible explanation for the synergistic action of inhibitors of the PI3K/mTOR pathways with inhibitors of glycolysis was recently found in cells derived from various cancer types [72]. These authors reported on an escape from glycolysis addiction of tumor cells by an mTORC1-dependent circumvention of the 2-DG-mediated glycolysis block via the pentose phosphate pathway back to glycolysis [72].

Combined treatment with 2-DG and SB203580 has recently been described in pancreatic and ovarian cell lines [73] with similar synergistic effects on cell survival in five of six cell lines as described here. On the other hand, Cheng et al. reported that the p38-MAPK pathway is necessary for apoptosis induced by 2-DG in pancreatic cancer cells [74]. Taken together, these data suggest that the effect of p38-MAPK inhibitors depends on the individual cell context and on activation pattern of signaling pathways within the cell.

Up to now, to the best of our knowledge, no data on the efficacy of a combined treatment with 2-DG and the c-MYC inhibitor or MEK inhibitor like PD98059 are available. Inhibition of c-MYC alone resulted in significant decrease of viable cells which was synergistically enhanced by 2-DG in our cells (Table 3). On the other hand, combination of the MEK inhibitor PD98059 with 2-DG showed a better effect than treatment with 2-DG alone (Table 3). Although we do not know the molecular reasons at this time, combined treatment of NHL cells with 2-DG and inhibitors of the PI3K/AKT pathway, c-MYC and p38 MAPK intracellular signaling pathways may be a promising new therapeutic option. Further experiments will provide further insights into the molecular background of cell inhibition and the mechanism of cell death induced by these substances.

## Conclusions

Taken together, our results suggest that PI3K/mTOR inhibitors as well as the c-MYC inhibitor decreased the viability of lymphoma cells, reduced glucose uptake and glucose metabolism, and downregulated the expression of glycolysis-associated genes and glucose metabolism-regulating miRNAs. Our results that 2-DG decreased viability of all cell lines support the hypothesis that lymphoma cells are highly dependent upon high glycolysis for survival. In combination with inhibitors of intracellular signaling pathways, 2-DG treatment may be an option to reduce proliferation of NHL cells.

## Abbreviations

2-DG: 2-deoxy-d-glucose
ABC: activated B cell like
AKT: protein kinase B
AML: acute myeloid leukemia
DLBCL: diffuse large B cell lymphoma
FBS: fetal bovine serum
FDG: ^18^F-fluoro-2-deoxy-d-glucose
G6Pase: glucose-6-phosphatase
GCB: germinal center B cell like
GLUTS: glucose transporters
HIF-1: hypoxia-inducible factor 1
HK: hexokinase
HPRT1: hypoxanthine phosphoribosyltransferase 1
LDHA: lactate dehydrogenase A
MAPK: mitogen-activated protein kinase
MEK: mitogen-activated protein kinase kinase
miRNAs: microRNAs
mTOR: mammalian target of rapamycin
NHL: non-Hodgkin’s lymphoma
PET: positron emission tomography
PI3K: phosphoinositide 3-kinase
PTEN: phosphatase and tensin homolog
